# Mucoadhesive Chitosan–Gellan Gum Nanoparticles for Rifampicin Delivery: Taguchi Optimization and In Vitro Release Behavior

**DOI:** 10.3390/pharmaceutics18050627

**Published:** 2026-05-21

**Authors:** Siu-Yin Cheung, Aldana Galiyeva, Yerkeblan Tazhbayev, Tolkyn Zhumagaliyeva, Yuliia Bardadym, Vladimir Aseyev

**Affiliations:** 1 Institute of Chemical Issues, Karaganda National Research University Academician Ye.A. Buketov, Karaganda 100028, Kazakhstan; cheung.s8103@gmail.com (S.-Y.C.); zhumagalievat79@mail.ru (T.Z.); 2 Department of Chemistry, Faculty of Science, University of Helsinki, 00014 Helsinki, Finland; yuliia.bardadym@helsinki.fi (Y.B.); vladimir.aseyev@helsinki.fi (V.A.)

**Keywords:** nanoparticles, chitosan, gellan gum, antituberculosis drug, polyelectrolyte complex coacervation, polysaccharide, rifampicin

## Abstract

**Background/Objectives:** Tuberculosis treatment remains challenging due to the limited stability and side effects of conventional rifampicin formulations. This study aimed to synthesize and optimize mucoadhesive chitosan–gellan gum nanoparticles for improved rifampicin delivery. The novelty of this work was the introduction of ethanol into the synthesis process, which improved the solubility of rifampicin and contributed to the formation of nanoparticles with the desired physicochemical characteristics. **Methods:** Rifampicin-loaded chitosan–gellan gum nanoparticles were produced using the polyelectrolyte complex coacervation method. The polymer ratios, drug-to-polymer ratio, temperature and ethanol volume were the main factors that were optimized using the Taguchi method. The physicochemical properties, such as TGA, DSC and FTIR spectroscopy, were investigated. In addition, drug release, mucoadhesive properties and mycobacterial activity against the H37Rv strain of *Mycobacterium tuberculosis* were examined. **Results:** Optimization using the Taguchi method produced nanoparticles with a narrow particle distribution (PDI: 0.212 ± 0.021), a satisfactory average size (153 ± 3 nm) and stability against aggregation (zeta potential: 22.94 ± 1.30 mV). A study of the degree of rifampicin release from nanoparticles showed that the drug release is influenced by pH and has a prolonged effect. Drug-loaded nanoparticles exhibited increased mucoadhesion compared with the pure drug. The minimum inhibitory concentration of rifampicin in chitosan–gellan gum nanoparticles for the suppression of the H37RV strain of *Mycobacterium tuberculosis* was determined. Spectroscopic and thermal analyses confirmed the incorporation of rifampicin in the polymer matrix. **Conclusions:** The developed chitosan–gellan gum nanoparticles represent a promising mucoadhesive delivery system for rifampicin. The incorporation of ethanol and the use of Taguchi optimization provide an effective strategy for controlling nanoparticle properties and improving drug delivery performance.

## 1. Introduction

Tuberculosis (TB) is a bacterial infection caused by *Mycobacterium tuberculosis* (MTB) and is one of the most common diseases in the world. The leading causes of death worldwide, based on the total number of deaths, are divided into two broad groups: cardiovascular diseases (such as coronary heart disease and stroke) and respiratory diseases (such as COVID-19, chronic obstructive pulmonary disease and lower respiratory tract infections), with tuberculosis ranking third among infectious diseases [1]. The total number of people who died from tuberculosis in 2024 was 1.23 million, of whom 150,000 were people living with human immunodeficiency virus [2]. Although the World Health Organization has developed a strategy to eradicate tuberculosis (aiming to reduce tuberculosis incidence by 50% and tuberculosis mortality by 75%), this has not yet been achieved, so more effective therapeutic measures need to be implemented [2]. Despite the availability of effective antituberculosis drugs, the treatment of this disease is a serious problem due to insufficient delivery of drugs to the sites of infection, low adherence to treatment, high dosages and treatment regimens, the emergence of drug resistance and serious side effects [3,4,5].

Traditional treatment for tuberculosis involves oral therapy, in which the patient takes the drug in the form of tablets, capsules or powder. First-line drugs such as isoniazid, rifampicin (RIF), pyrazinamide and ethambutol are used to treat tuberculosis in its early stages and in cases of primary infection [6]. However, with a high bacterial load, achieving and maintaining therapeutically significant concentrations in lung tissue and alveolar macrophages can be challenging, usually requiring higher doses. This, in turn, predisposes patients to hepatotoxicity and other undesirable side effects, impairs patients’ quality of life, and reduces their adherence to treatment [6]. In addition, suboptimal antibiotic concentrations contribute to the development of drug-resistant strains of *Mycobacterium tuberculosis*, which significantly complicates treatment [3,7,8].

In this regard, a relevant task for modern pharmaceuticals and nanomedicine is to develop new strategies for delivering antituberculosis drugs aimed at improving treatment efficacy and reducing systemic toxicity. In recent years, controlled delivery systems based on biodegradable polymers that are capable of providing prolonged and dose-controlled release of drugs have attracted particular interest [9,10,11]. Such systems make it possible to reduce the frequency of drug administration, decrease the total dose and, as a result, reduce the risk of side effects [12].

Natural polymers, particularly polysaccharides, are widely used for drug delivery due to their biocompatibility, biodegradability, low immunogenicity, and non-toxicity [13,14,15]. Chitosan (CS), the second most abundant polysaccharide in nature after cellulose, is a bioavailable and mucoadhesive polymer that is widely used for producing nanoparticles for the delivery of antituberculosis drugs [16,17,18]. Chitosan is often combined with other polysaccharides, such as alginate, gellan gum (GG) and guar gum, resulting in improved physicochemical and biological properties [19,20]. The inclusion of anionic polysaccharides increases the stability of nanoparticles, improves mucoadhesion, and ensures controlled drug release while maintaining the high biocompatibility of the delivery system [21,22,23].

Although there are some works on the development of chitosan–gellan gum nanoparticles, some important parameters, such as loading efficiency and nanoparticle yield, remain insufficiently investigated in previous works [21,22,23,24]. To date, no work on the development of chitosan–gellan gum nanoparticles loaded with rifampicin has been reported, making this study a novel contribution to the field of the delivery of antituberculosis drugs. Moreover, ethanol was added during the synthesis process, which improves the solubility of rifampicin and contributes to the formation of nanoparticles with desired physicochemical characteristics. Optimizing particle size further improves their distribution in the lower respiratory tract [25]. Various methods are used to optimize and minimize experiments, such as the Taguchi method, central composite design, and the Box–Behnken design [26,27,28]. In our study, we used the Taguchi method to optimize the synthesis of chitosan–gellan gum nanoparticles for loading rifampicin (CS-GG-RIF NPs). Parameters such as the GG/CS ratio, the RIF/polymer ratio, temperature and the volume of ethanol were studied. To improve the solubility of rifampicin, increase the drug-loading efficiency, and minimize the size of nanoparticles, we used ethanol for the first time to produce chitosan–gellan gum nanoparticles by the polyelectrolyte complex coacervation method. In addition, the important physicochemical properties of the produced nanoparticles, the degree of drug release from the polymer matrix, and the mucoadhesive and antimycobacterial properties of the nanoparticles were investigated.

## 2. Materials and Methods

### 2.1. Materials

Chitosan (low molecular weight; 50–190 kDa; ≥75% deacetylated), gellan gum (GelzanTM CM; M = 1000 kg/mol), rifampicin (≥97%; HPLC) and fluorescein isothiocyanate (FITC) isomer I (≥90%; HPLC) were purchased from Sigma–Aldrich (Taufkirchen, Germany). Ethanol (90%; produced by Dosfarm, Almaty, Kazakhstan) was purchased from a pharmacy.

### 2.2. Purification of Chitosan

The purification of chitosan followed the method reported by Qian et al., with modifications [29,30]. Pale-yellow chitosan powder (0.500 g) was dissolved in 0.1 M HCl (50 mL) by shaking the suspension overnight at 40 °C (Biobase Orbital Shaker-Incubator BK-CIS20, Jinan, China). Insoluble particles were removed from the solution by filtration with cotton wool and then centrifugation at 10,400 rpm for 1 h (Eppendorf 5420, Eppendorf SE, Hamburg, Germany). The supernatant was titrated by dropwise addition of 0.5 M NaOH with stirring until the pH reached 10. Purified chitosan was obtained by centrifugation (7300 rpm for 30 min) and washed with distilled water 3 times (7300 rpm for 10 min each time). Lyophilization (Martin Christ Alpha 1-2 LDplus, Osterode am Harz, Germany) of the pellet produced 0.455 g of dried white chitosan (yield of purification = 91%).

### 2.3. Purification of Gellan Gum

The purification of gellan gum followed the method reported by Lavikainen et al., with modifications [31]. Pale-yellow gellan gum powder (0.300 g) was dissolved in deionized water (30 mL) by shaking the suspension overnight at 60 °C. The solution was filtered through cotton wool and centrifuged at 5000 rpm for 30 min (CENLEE 4K, Changsha, China). The supernatant was precipitated in acetone (75 mL). Purified white gellan gum (0.245 g) was collected by vacuum filtration and dried by lyophilization (yield of purification = 81.7%).

### 2.4. Preparation of Chitosan and Gellan Gum Stock Solutions

The chitosan solution (1 mg/mL) was prepared by the addition of purified chitosan in 0.1 M hydrochloric acid and stirred overnight at 40 °C. The gellan gum solution (1 mg/mL) was prepared by stirring the purified gellan gum in deionized water at 80 °C for 1 h and then overnight at 60 °C. The gellan gum solution was filtered by cotton wool prior to use.

### 2.5. Preparation of Chitosan–Gellan Gum Nanoparticles with Rifampicin (CS-GG-RIF NPs)

The preparation of nanoparticles followed the method of chitosan–gellan gum gel preparation, with modifications [32]. Different mass ratios of gellan gum to chitosan were mixed (1:9, 4:6 or 7:3). Both solutions were adjusted to pH 5 by adding 0.1 M HCl or 0.2 M NaOH in advance. The gellan gum solution was added to the chitosan solution dropwise (at 25 °C, 40 °C or 60 °C). After stirring for 15 min, rifampicin (rifampicin: polymers = 1:3, 1:1 or 3:1) in ethanol (6 mg/mL) was added dropwise, followed by adding 90% ethanol dropwise (total volume of ethanol = 5 mL, 10 mL or 15 mL). The reaction mixture was stirred for 2 h, and the temperature was decreased from 60 °C (or 40 °C) to 25 °C simultaneously. Then, the reaction mixture was taken for the measurements of particle size, polydispersity and zeta potential. The nanoparticles were then isolated by centrifugation at 15,060 rpm (Eppendorf 5420, Hamburg, Germany) for 30 min and rinsed with distilled water by three cycles of centrifugation for 5 min each. The pellet was collected and dried.

### 2.6. Design of Taguchi Experiment

The Taguchi design of experiments method was applied in this study to evaluate the impacts of various parameters on the optimization and reduction in the number of experiments. This method is suitable for studying multiple factors using the Design-Expert software (version 13, Stat-Ease, Minneapolis, MN, USA). We evaluated four factors, including the GG/CS ratio, the RIF/polymer ratio, temperature and the volume of ethanol by using the L9 orthogonal matrix (Table 1).

### 2.7. Preparation of FITC–Chitosan

The preparation procedure followed the methods reported by Cook et al. and Ge et al., with modifications [33,34]. To the chitosan solution (1% *w*/*v* in 0.1 M acetic acid; 1 mL), ethanol (1 mL) and FITC (2 mg/mL; 0.5 mL) were added, and the reaction mixture was stirred in the dark at 25 °C for 3 h, followed by precipitation in NaOH (0.1 M; 10 mL). FITC–chitosan was isolated by centrifugation at 15,060 rpm (Eppendorf 5420, Hamburg, Germany) for 30 min and washed with distilled water (15,060 rpm; 10 min each) until no fluorescence was detected in the supernatant by using a fluorescence microscope (AOSVI, Shenzhen, China). The pellet was re-dissolved in 3 mL of 0.1 M acetic acid and dialyzed (MwCO: 8000–14,000 D, Lombard, IL, USA) in 50 mL of distilled water for 3 days in the dark, with the water being replaced every day. The dialyzed product was dried by lyophilization.

### 2.8. Preparation of FITC–Chitosan–Gellan Gum Nanoparticles with Rifampicin (FITC-CS-GG-RIF NPs)

The preparation was carried out according to the method reported by Cook et al., with modifications [33]. Both chitosan and gellan gum solutions were adjusted to pH 5 by adding 0.1 M HCl or 0.2 M NaOH in advance. An amount of 0.52 mg of fluorescein isothiocyanate (FITC) (in 0.5 mL of ethanol) was added dropwise to 3.6 mL of the chitosan solution in the dark at 25 °C for 4 h. An amount of 2.4 mL of the gellan gum solution was added to the reaction mixture dropwise at 40 °C. After stirring for 15 min, 2 mg of rifampicin in ethanol (6 mg/mL) was added dropwise, followed by adding 90% ethanol dropwise (total volume of ethanol = 15 mL). The reaction mixture was stirred for 2 h in the dark, and the temperature was decreased from 40 °C to 25 °C simultaneously. Then, the nanoparticles were isolated by centrifugation at 15,060 rpm (Eppendorf 5420, Hamburg, Germany) for 30 min and rinsed with distilled water by four cycles of centrifugation for 10 min each. The absence of free FITC in the supernatant was confirmed by fluorescence microscopy. The pellet was collected and dried by lyophilization.

### 2.9. Measurement of Particle Size, Polydispersity, Zeta Potential and Transmission Electron Microscopy

The average diameter, polydispersity index (PDI) and zeta potential of the nanoparticles were measured by a Zetasizer Advance Pro (Malvern Panalytical Ltd., Worcestershire, UK). Ten drops of the nanoparticle suspension were added to 1.5–2 mL of distilled water in a quartz cuvette. The dynamic light scattering measurements were carried out at 25 °C with a 173° angle determination. The zeta potentials were determined by adding 20 drops of the nanoparticle suspension to 1 mL of distilled water in a folded capillary cell. Transmission electron microscopy (TEM) images were examined using a Hitachi S-4800 field-emission scanning electron microscope (Hitachi High-Tech, Hitachinaka, Japan) at an accelerating voltage of 30 kV. TEM grids were prepared by placing 10 µL of the diluted sample solutions on a carbon-coated copper grid and allowing the solution to evaporate completely at room temperature.

### 2.10. Determination of the Yield of Nanoparticles, Drug Encapsulation Efficiency and Drug-Loading Degree

The centrifugation of nanoparticle suspensions was carried out after the synthesis. The pellet was dried, and the yield of nanoparticles was determined:

$$Yield\;of\;nanoparticles\left(\%\right)=\frac{mass\;of\;nanoparticles}{total\;mass\;of\;polymers+total\;mass\;of\;drugs}\times 100\%$$

In addition, the supernatant was taken to determine the amount of free drugs by UV–visible spectroscopy (Promecolab PE-5400UV, Shanghai Mapada Instruments Co., Ltd., Shanghai, China). The encapsulation efficiency (EE) was calculated according to the formula below.

$$Encapsulation\;efficiency\left(\%\right)=\frac{total\;mass\;of\;drug-mass\;of\;free\;drug}{total\;mass\;of\;drug}\times 100\%$$

### 2.11. Thermogravimetric Analysis (TGA) and Differential Scanning Calorimetry (DSC)

Thermogravimetric and differential scanning calorimetry analyses were performed with a LabSYS evo TGA/DTA/DSC analyzer (Setaram Instrumentation Caluire-et-Cuire, France). The temperature spectrum was varied from 30 to 700 °C, employing an aluminum oxide crucible. The sample was heated at 10 °C/min in a nitrogen atmosphere at a flow rate of 30.

### 2.12. Fourier-Transform Infrared Spectroscopy

The samples were analyzed by infrared spectroscopy with an FSM 1202 spectrometer (Infraspek Ltd., Saint Petersburg, Russia). Fourier-transform infrared (FTIR) spectra were obtained by using the KBr method. A pellet was prepared by blending around 3 mg of the sample with 100 mg of KBr. The scanning range was set from 4000 to 500 cm^−1^, with a resolution of 8 cm^−1^.

### 2.13. In Vitro Release of Drug from Nanoparticles

The release behavior of rifampicin from nanoparticles was examined at pH 7.4 using different rifampicin-to-polymer ratios (1:3, 1:1, and 3:1). The effect of pH (1.2, 6.8, and 7.4) was further evaluated for nanoparticles with a rifampicin-to-polymer ratio of 3:1. Samples of nanoparticles containing the same amount of rifampicin were put into dialysis membranes (MwCO: 8000–14,000 D, Lombard, IL, USA), followed by adding 2.5 mL of dilute HCl (pH 1.2), phosphate buffer solution (PBS) (pH 6.8) and PBS (pH 7.4), respectively. They were immersed in 100 mL of dilute HCl (pH 1.2), PBS (pH 6.8) and PBS (pH 7.4), separately, at 37 ± 0.5 °C, which was regulated by a circulating water bath (SIA “ELMI”, Riga, Latvia). The portions of the medium (2 mL) were taken at predetermined times. The extracts were analyzed by UV–visible spectroscopy at 470 nm.

### 2.14. Mucoadhesion Study of CS-GG-RIF Nanoparticles

Samples of FITC–chitosan, FITC-CS-GG-RIF and rifampicin were separately applied to the surfaces of three ovine lung tissue samples [35]. The ovine lung tissue was cut into pieces of 2 × 2 cm^2^ and was mounted on a glass slide. The samples were mounted on microscope slides, inclined at 45° and rinsed with phosphate buffer solution (pH 7.4) at 37 °C using a flow rate of 2 mL/min until a total volume of 500 mL was delivered. Each experiment was conducted for more than 4 h. After every 100 mL of buffer had been applied, the amounts of the samples remaining on the lung tissue were determined. FITC–chitosan and FITC-CS-GG-RIF were analyzed by fluorescence microscopy under blue light with the software ImageJ 1.54m, while rifampicin was quantified by measuring its amount in the rinsate using UV–visible spectroscopy. The results of the FITC–chitosan and FITC-CS-GG-RIF were both expressed as the normalized intensity, whereas rifampicin retention was reported as the percentage of mass retained.

### 2.15. Determination of Antimycobacterial Activity of CS-GG-RIF Nanoparticles

In vitro studies were performed in Airstream AC2-4E8 biosafety cabinets (Esco Micro Pte. Ltd., Singapore), which provided sterile conditions through negative pressure and air filtration through HEPA filters. The antimycobacterial activity of the nanoparticles was evaluated using an antituberculosis-sensitive wild strain of MTB H37Rv, obtained from the Asklepios pulmonology clinic (Munich-Hauting, Germany). Determination of mycobacterial activity was carried out on a dense Löwenstein–Jensen medium. CS-GG NPs were synthesized with rifampicin, and the drug concentrations were 0.2 mg/mL, 0.5 mg/mL and 1 mg/mL. Blank CS-GG NPs without the drug were used as a control. All tubes of each experimental row were seeded with 0.2 mL of a bacterial suspension. The tubes were sealed with silicone stoppers and incubated in the thermostat at 37 °C for 21 days. The results of suppressive activity were evaluated by counting the number of colony-forming units (CFUs) of MTB on the nutrient medium and comparing the control with the experimental cultures. For samples showing positive results, smears were made and stained according to the Ziehl–Neelsen method [36]. The smear was allowed to air-dry and examined microscopically using a Leica DMLS (Wetzlar, Germany) binocular microscope.

### 2.16. Statistical Processing of the Produced Data

All studies were carried out at least three times. The results are presented as mean values with standard deviations. In order to compare the independent groups, a one-way ANOVA was performed using the OriginPro 2019b software.

The effects of the proposed experiments on responses were analyzed using the Design-Expert 13 software to obtain the main effects of different factors independently, and then an analysis of variance (ANOVA) was performed to determine statistically significant factors. The optimal conditions were determined by selecting the function.

## 3. Results

### 3.1. Synthesis of CS-GG-RIF Nanoparticles and Optimization Using the Taguchi Method

Polysaccharide nanoparticles can be synthesized using various methods, including ionotropic gelation, complex coacervation, nanoprecipitation, and others [37,38,39,40,41,42]. Table 2 presents a summary of representative rifampicin-loaded polysaccharide nanoparticles.

**Figure 1:**
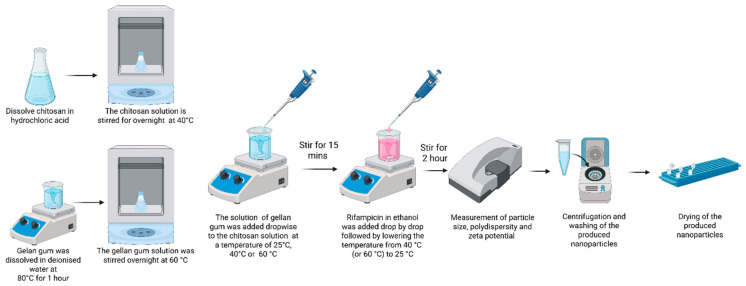
Schematic representation of CS-GG-RIF NP production by polyelectrolyte complex coacervation method. This image was created using BioRender.

In this study, the polyelectrolyte complex coacervation method was used (Figure 1), which does not require expensive equipment or the use of additional cross-linking or stabilizing agents. Chitosan–gellan gum-based nanoparticles are mostly used for encapsulating hydrophilic drugs [37,45], but there are also examples in the literature of such systems being used for hydrophobic drugs [37,46]. CS-GG-RIF nanoparticles were produced using the previously developed technique for producing CS-GG gel, with modifications [32]. Owing to the large size (2286 ± 27) and the broad PDI value (0.958 ± 0.059) of the prepared particles, the preparation method needs to be optimized for the production of nanoparticles. Schnell et al. reported that the size of xylan/chitosan nanoparticles decreased from 1055 nm to 651 nm as the ethanol concentration was increased from 0 wt% to 16 wt, which could be due to the decrease in the ionic charges in the system [47]. In our work, we used ethanol to load the hydrophobic drug rifampicin, which helped increase the encapsulation efficiency. In this study, for the first time, ethanol was used in the synthesis of CS-GG nanoparticles by the polyelectrolyte complex coacervation method. Ethanol not only improves the solubility of rifampicin but also contributes to the formation of nanoparticles with smaller sizes and a narrower size distribution.

The aim of this study was to optimize the production of nanoparticles based on chitosan and gellan gum loaded with rifampicin. Some factors affecting the nanoparticle preparation processes were analyzed: the GG/CS ratio, the RIF/polymer ratio, temperature and the volume of ethanol. The physicochemical properties of the produced NPs were studied, and the in vitro release of rifampicin was determined.

We used a Taguchi orthogonal array to determine the optimal conditions and identify the key parameters affecting the size, polydispersity index (PDI), zeta potential, encapsulation efficiency of the drug and yield of nanoparticles. CS-GG-RIF nanoparticles were synthesized using the polyelectrolyte complex coacervation method. We performed nine experiments to determine the best synthesis conditions for the nanoparticles. Table 3 shows the structure of the orthogonal matrix and the results of the physicochemical characteristics. The particle size of each nanoparticle was measured using dynamic light scattering, the zeta potential was measured using mixed-mode measurement phase analysis light scattering, and the drug encapsulation efficiency and nanoparticle yield were calculated.

According to the obtained results, depending on the synthesis conditions, chitosan–gellan gum particles with an average particle size ranging from 151 ± 3 nm to 836 ± 19 nm can be produced, and the polydispersity varies significantly from 0.221 ± 0.014 to 0.683 ± 0.113. The zeta potential of the produced particles was positive and ranged from 17.80 ± 0.72 mV to 32.48 ± 0.53 mV. In addition, the encapsulation efficiency of rifampicin and the nanoparticle yield ranged from 20% to 73% and from 10% to 65%, respectively.

The particle size, polydispersity, zeta potential, drug encapsulation efficiency and nanoparticle yield data of CS-GG-RIF NPs were analyzed using the Design-Expert software. An ANOVA table was used to determine the key process parameter affecting the properties of CS-GG-RIF NPs. The ANOVA tables for the Taguchi method are presented in Table 4. For all nanoparticle characteristics, the initial response values were converted using the following equation: y′= (y + k)^λ^, where constant (k) = 0, and lambda (λ) = −1.24 for size, −1.78 for the PDI, and 0.8 for the zeta potential. The best lambda was predicted using the Design-Expert program.

*p*-values of less than 0.05 indicate the significance of the model terms. According to the ANOVA results, the GG/CS ratio and temperature did not have a significant effect on the average particle size, and these parameters, as well as the ethanol volume, did not affect the polydispersity. According to the results of the ANOVA analysis, none of the factors had a significant effect on the zeta potential.

**Figure 2:**
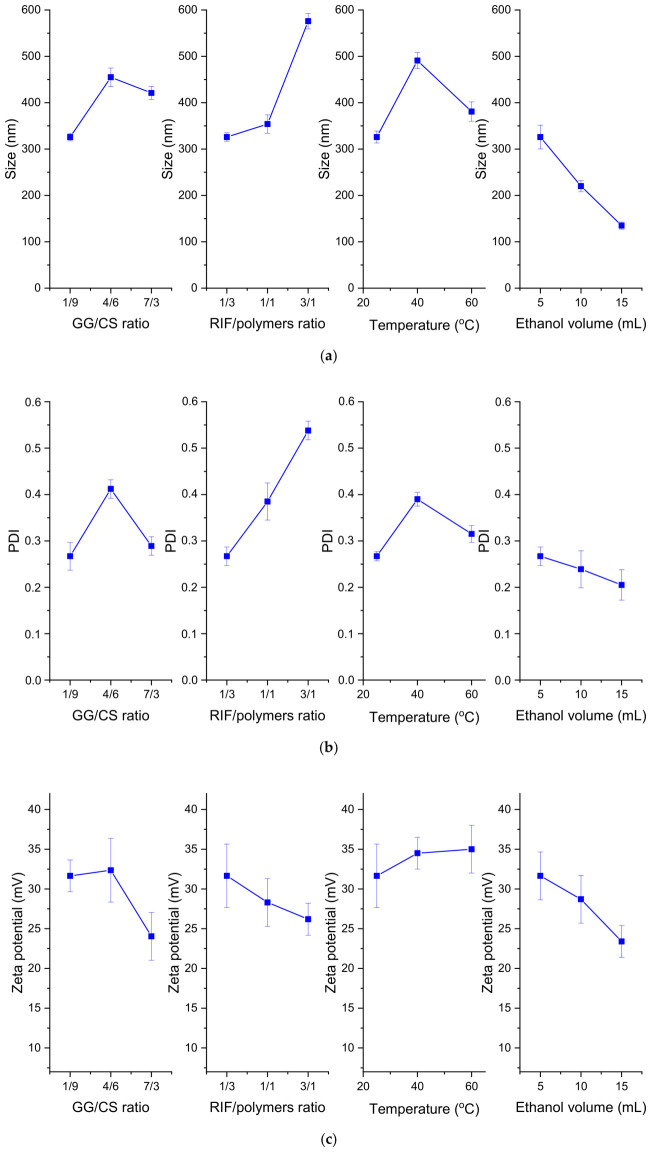
Effects of parameters on (**a**) average particle size, (**b**) PDI and (**c**) zeta potential of CS-GG-RIF NPs. Data are presented as means ± standard deviations (*n* = 3).

As we can see from the graphs presented in Figure 2, as the ratio of drug to polymer increased, the average size and PDI of the nanoparticles increased, which may be due to the inclusion of rifampicin in the space of the polymeric nanoparticles [10]. As the volume of ethanol increased, the average size and PDI of the nanoparticles decreased. This is explained by the fact that ionic charges decreased, thereby strengthening the interactions between polysaccharides [47,48]. As the temperature increased, the size of the nanoparticles increased, with the largest particle size and polydispersity occurring at 40 °C.

When the gellan gum content in the GG/CS ratio increased (Figure 2), the zeta potential of the particles decreased generally, which can be explained by the fact that the negative charges of gellan gum neutralize part of the positive charges of chitosan [22]. In addition, as the amount of ethanol increased, the zeta potential decreased, which may be due to the fact that ethanol solution has a higher viscosity and lower dielectric permeability than water, affecting, therefore, the calculation of the zeta potential based on electrophoretic mobility [49,50].

The encapsulation efficiency and nanoparticles yield are also important parameters, so we performed an ANOVA analysis, the results of which are presented in Table 5. The formula y′= (y + k)^λ^ was also used for the calculation, where constant (k) = 0, and lambda (λ) = −2.88 for EE and −0.55 for NPs’ yield.

**Figure 3:**
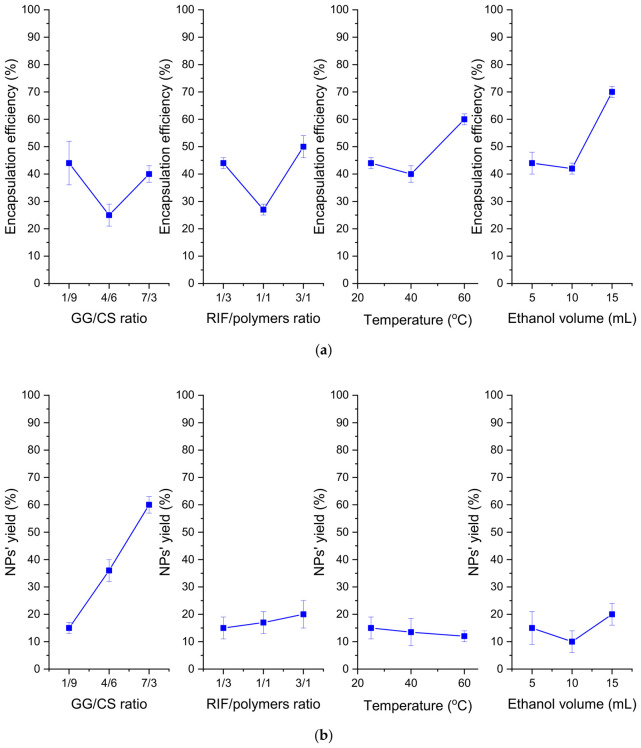
Effects of parameters on (**a**) encapsulation efficiency and (**b**) nanoparticle yield of CS-GG-RIF NPs. Data are presented as mean ± standard deviations (*n* = 3).

The encapsulation efficiency was influenced by the GG/CS ratio, RIF/polymer ratio, and temperature, and the model for nanoparticles yield was significant when the GG/CS ratio and volume of ethanol parameters were included. Thus, it can be concluded that both factors influence the properties of nanoparticles. The highest encapsulation efficiency was observed at a RIF/polymer ratio of 3/1 and an ethanol volume of 15 mL (Figure 3). This is due to the hydrophobicity of rifampicin in water and its good solubility when ethanol is used as a co-solvent [51]. Figure 3 shows that the highest nanoparticle yield was observed at a GG/CS ratio of 7/3 and an increase in the ethanol volume.

Based on the ANOVA results, we performed optimization using the Design-Expert software for the synthesis of CS-GG-RIF NPs. We applied criteria (Table 6) to optimize the synthesis process for nanoparticles with the minimum size and polydispersity, as well as the maximum zeta potential, encapsulation efficiency and particle yield. According to the ANOVA results, the minimum size and PDI value were theoretically predicted at 25 °C (Figure 2). Therefore, the temperature for optimization was set at 25 °C.

Among the options proposed by the program, the solution with the highest desirability of 62.5% was selected, which provides satisfactory nanoparticle characteristics. Next, we carried out the synthesis under the proposed optimal conditions: a GG/CS ratio of 4/6, an RIF/polymer ratio of 1/3, a temperature of 25 °C, and a volume of ethanol of 15 mL. The experimental results obtained were in reasonable agreement with the model (Table 7). There was a difference between the predicted and experimental values, but according to the model, it was within the desirability limit (the error was no more than 62.5%). It is crucial to note that nanoparticles with a higher encapsulation efficiency and nanoparticle yield can be produced under alternative conditions, but this leads to an increase in particle size and polydispersity. Therefore, the selected parameters enable the synthesis of nanoparticles with a smaller size, a narrower size distribution and better colloidal stability, as these characteristics are crucial for mucoadhesive drug delivery systems. Thus, it can be concluded that the Taguchi method is suitable for optimizing the synthesis conditions for producing CS-GG-RIF NPs.

### 3.2. Study of the Physicochemical Properties of the Produced CS-GG-RIF Nanoparticles

**Figure 4:**
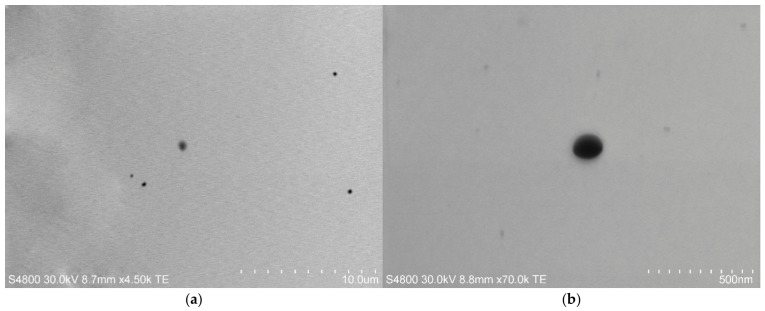
TEM images of CS-GG-RIF nanoparticles. Scale bars are (**a**) 10 µm and (**b**) 500 nm.

To determine the morphology of the produced CS-GG-RIF nanoparticles, transmission electron microscopy (TEM) was performed (Figure 4). Using the ImageJ software, the average size of the nanoparticles was calculated as 137 ± 11 nm. The produced CS-GG-RIF particles had a spherical shape.

**Figure 5:**
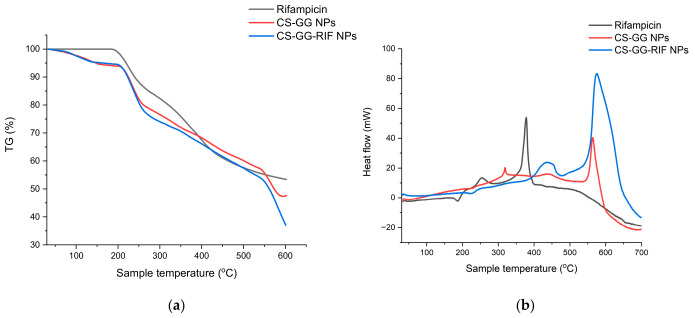
(**a**) TGA and (**b**) DSC analysis results for rifampicin, CS-GG NPs and CS-GG-RIF NPs.

The TGA and DSC curves of rifampicin, CS-GG nanoparticles without the drug, and rifampicin-loaded CS-GG-RIF nanoparticles are shown in Figure 5. The results showed a slower rate of decomposition of rifampicin-loaded chitosan–gellan gum nanoparticles compared with empty chitosan–gellan gum nanoparticles, indicating improved stability. The results show that the particles begin to disintegrate at a temperature of 86 °C; after 200 °C, a sharp decrease in weight was observed, which may be associated with the degradation of polysaccharides [52,53]. The endothermic peak characteristic of rifampicin was marked by a change in enthalpy at a temperature of 186.6 °C (Figure 5b), which corresponded to its melting point, i.e., reflecting the crystalline nature of rifampicin. Next, an exothermic peak was observed at a temperature of 378.3 °C, in the range of 188–550 °C, with a weight loss of up to 45% (Figure 5a), which may correspond to the decomposition temperature of the drug. There was no endothermic peak for rifampicin in the thermogram of CS-GG-RIF nanoparticles, which indicates the formation of an amorphous state or molecular dispersion of the drug in the matrix.

**Figure 6:**
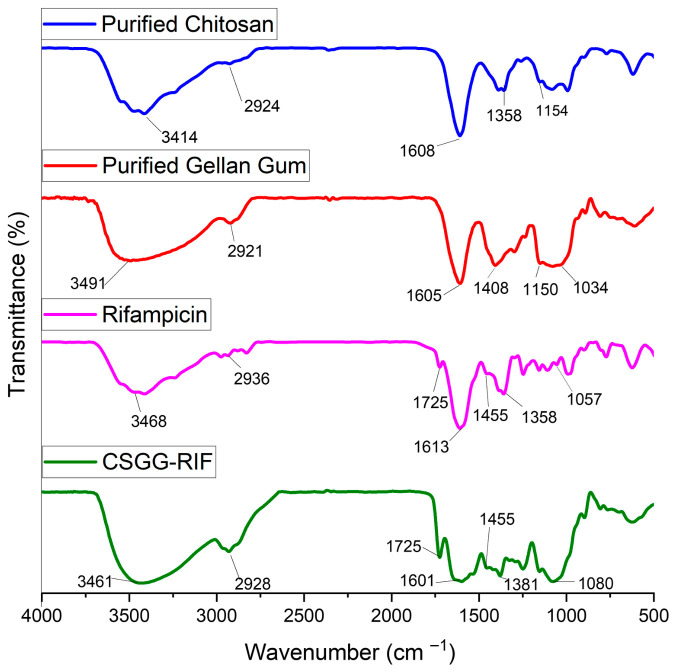
FTIR spectra of purified chitosan, purified gellan gum, rifampicin and CS-GG-RIF nanoparticles.

To determine the incorporation of rifampicin into the polymer matrix of chitosan and gellan gum, FTIR spectroscopy studies were conducted for the initial components of the system and the synthesized nanoparticles. The results are presented in Figure 6.

For purified chitosan, the absorption band at 3414 cm^−1^ corresponds to the N–H group in primary amines and O–H in the pyranose ring, 2924 cm^−1^ for C–H stretching, 1608 cm^−1^ for C = O stretching and 1358 cm^−1^ for CH_3_ in NHCOCH_3_, and 1154 cm^−1^ for glycosidic linkage C–O–C [54,55]. The spectra of pure gellan gum are characterized by broad and intense peaks at 3491 cm^−1^, which are associated with O–H stretching, at 2921 cm^−1^ for C–H vibrations, at 1605 cm^−1^ and 1408 cm^−1^ for symmetric and asymmetric valence vibrations of COO^−^, and at 1150 cm^−1^ and 1034 cm^−1^ associated with C–O stretching [56,57]. The spectra of pure rifampicin show broad and intense peaks at 3468 cm^−1^, which are associated with N–H stretching, and C–H stretching vibrations are noticeable in the range of 2936 cm^−1^. Bands at 1725 cm^−1^ and 1613 cm^−1^ correspond to C = O and C = N stretching, respectively; 1455 cm^−1^ is due to C = C stretching, 1358 cm^−1^ is associated with CH_2_ and C = C, and 1059 cm^−1^ corresponds to –CH, CO, and C–H [10,58,59]. The graph for CS-GG-RIF nanoparticles shows peaks corresponding to both polymers and drugs, confirming the inclusion of rifampicin in the polymer matrix without chemical interaction.

### 3.3. Study of Rifampicin Release from the Polymer Matrix In Vitro

**Figure 7:**
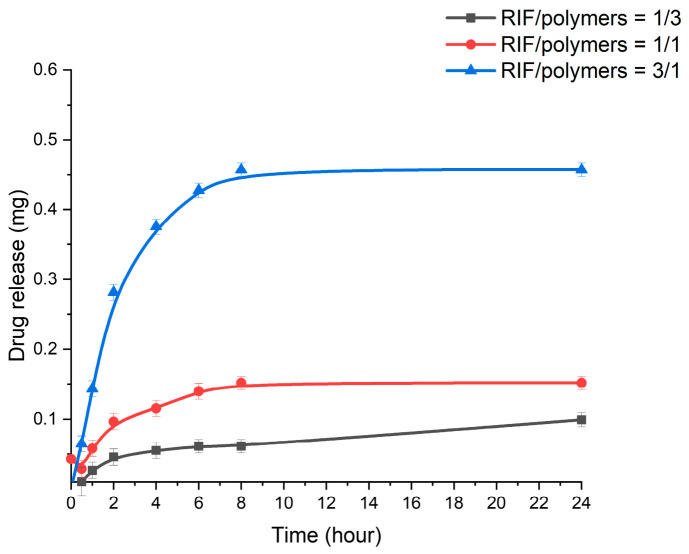
Drug release from CS-GG-RIF NPs with different rifampicin/polymers ratios. Data are presented as means ± standard deviations (*n* = 3).

Rifampicin release from the polymer matrix was studied by dialysis at 37 °C and pH 7.4. Nanoparticles with different RIF/polymer ratios (1/3, 1/1, and 3/1) were studied. The amount of rifampicin loaded into the polymers was the same, and the results are presented in Figure 7. At the beginning of the process, there was a rapid release of the drug adsorbed on the surface of the nanoparticles. The polymer then swelled, causing the drug to be gradually released from the polymer matrix [60]. As the RIF/polymer ratio increased from 1/3 to 3/1, the amount of release tended to increase from 0.1 mg to 0.45 mg after 24 h. The higher the drug loading, the higher the concentration of rifampicin in the polymer matrix. This enhanced the driving force of diffusion, and the drug was released more rapidly from the chitosan–gellan gum nanoparticles [60,61].

**Figure 8:**
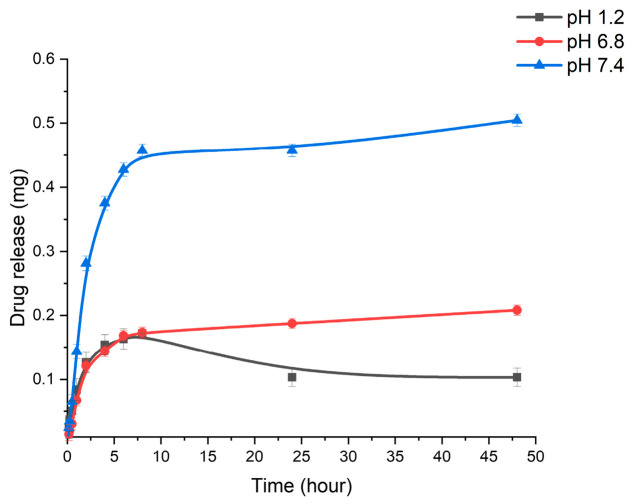
Cumulative drug release from CS-GG-RIF NPs (RIF/polymers = 3/1) at different pH values. Data are presented as means ± standard deviations (*n* = 3).

In addition, the degree of drug release depended on the medium’s pH. Thus, we studied the release of rifampicin in a RIF/polymer ratio of 3/1 at different medium pH values (pH 1.2, 6.8, and 7.4). The curves (Figure 8) show that the medium pH affected the release of rifampicin from chitosan–gellan gum nanoparticles. After 48 h of ± study, the content of rifampicin released from CS-GG nanoparticles at pH 7.4, 6.8, and 1.2 corresponded to 0.5 mg, 0.2, and 0.1 mg, respectively. Rifampicin was released faster in a neutral environment compared with an acidic environment, which may be due to electrostatic interactions between oppositely charged polymers. In an acidic environment, chitosan is more protonated and attracted to gellan gum, which leads to a decrease in swelling and a slowdown in drug release. At a neutral pH, weakened electrostatic interactions contribute to the relaxation of the matrix and accelerate the diffusion of the drug.

It can be concluded that the in vitro release of rifampicin from chitosan–gellan gum nanoparticles was prolonged, which makes it possible to use the produced particles for the delivery of antituberculosis drugs.

The rifampicin release kinetics from chitosan–gellan gum nanoparticles were analyzed using mathematical models, including zero-order (Q_t_ = Q_0_ + k_0_t), first-order (dC/dt = −kC), Higuchi (Mt/M∞ = k√t) and Korsmeyer–Peppas (Mt/M ∞ = ktn) [62,63,64] to determine the mechanism of drug release from the developed polymeric system (Table 8).

The zero-order and Higuchi models demonstrated moderate applicability under various pH conditions (1.2, 6.8 and 7.4). The release of rifampicin from chitosan–gellan gum nanoparticles is not strictly governed by zero-order kinetics and is not controlled by simple Fickian diffusion through a homogeneous matrix. The Korsmeyer–Peppas model showed high correlation coefficients (R^2^ = 0.9149–0.9705) at all pH values, indicating that the mechanism of rifampicin release is a combined process involving diffusion and polymer degradation. At pH 6.8 and 7.4, the first-order model showed excellent correlation (R^2^ = 0.9802 and 0.9976, respectively). Thus, under neutral conditions, the nanoparticles behave more predictably. During rifampicin release, processes of diffusion and swelling of the polymer matrix occur. Overall, the kinetics of rifampicin release from chitosan–gellan gum nanoparticles depend on pH. This behavior is desirable for oral delivery systems, as it can help protect the drug from premature release in the stomach and ensure prolonged release in the intestine.

### 3.4. Investigation of the Mucoadhesive Properties of CS-GG-RIF NPs

Antituberculosis drugs are administered orally, which is often associated with low bioavailability and side effects [65,66,67], whereas inhalation delivery to the lungs is preferable but requires overcoming biological barriers [10]. A promising solution is the use of mucoadhesive polymeric nanoparticles based on chitosan, which are capable of interacting with mucin and improving drug retention [68,69].

**Figure 9:**
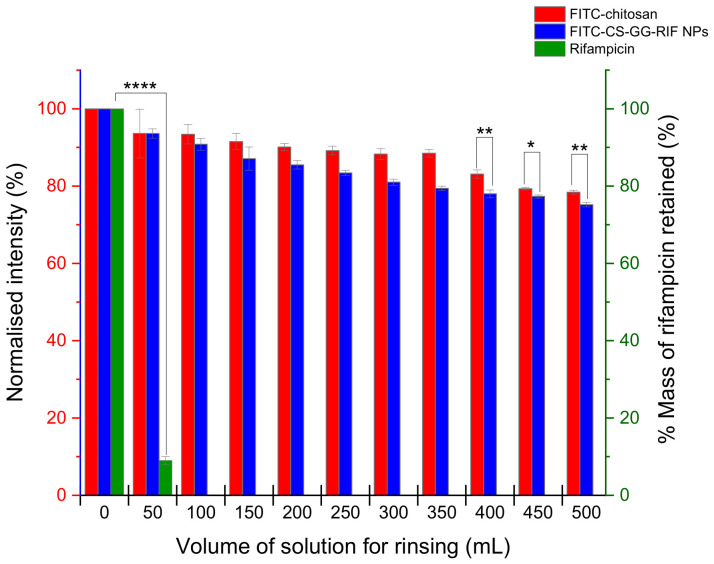
Retention of rifampicin, chitosan and CS-GG-RIF nanoparticles in the ovine lung tissue after washing with phosphate buffer solution (pH 7.4) at various volumes. Data are presented as means ± standard deviations (*n* = 3). Statistically significant differences are indicated as follows: * *p* < 0.05; ** *p* < 0.01; and **** *p* < 0.0001. The red and blue columns share the same *Y*-axis scale.

The mucoadhesive properties of rifampicin, chitosan and CS-GG-RIF nanoparticles containing fluorescein isothiocyanate were examined using a modified fluorescence flow-through retention test developed earlier by the Khutoryanskiy group [35]. The lungs were washed with 500 mL of phosphate buffer solution (pH 7.4; 37 °C) at a rate of 2 mL/min. For pure rifampicin (Figure 9), the percentage of mass after washing was determined using UV spectroscopy. As a result, rapid removal of rifampicin from the surface of the ovine lung tissue was observed during the first 25 min.

**Figure 10:**
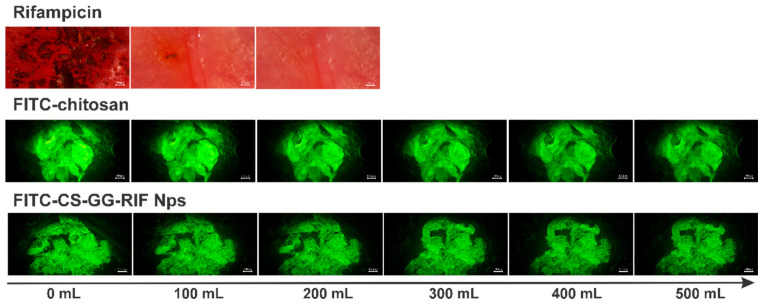
Fluorescent images illustrating the adhesion of polymeric materials to the ovine lung tissue washed with phosphate buffer solution (pH 7.4) at various volumes. Scale bar is 100 μm.

The retention profiles observed for chitosan and CS-GG-RIF nanoparticles in the ovine lung tissue were analyzed through a series of captured fluorescent photomicrographs, which are shown in Figure 10. Using the ImageJ software, the intensity was calculated, confirming that the nanoparticles could remain in the lung tissue for more than 4 h (Figure 9). Chitosan demonstrated a high retention capacity (78%), while the CS-GG-RIF nanoparticles exhibited a comparable level of mucoadhesion (75%). This suggests that the inclusion of rifampicin with gellan gum does not result in a significant loss of the system’s adhesive properties.

Chitosan and gellan gum were selected for their ability to improve the mucoadhesiveness of rifampicin and to increase the duration of drug release [70,71]. This minimizes washout effects, leading to improved patient compliance and a reduced frequency of administration, which is beneficial in drug delivery systems [72,73].

### 3.5. Investigation of the Mycobacterial Activity of CS-GG-RIF NPs

**Figure 11:**
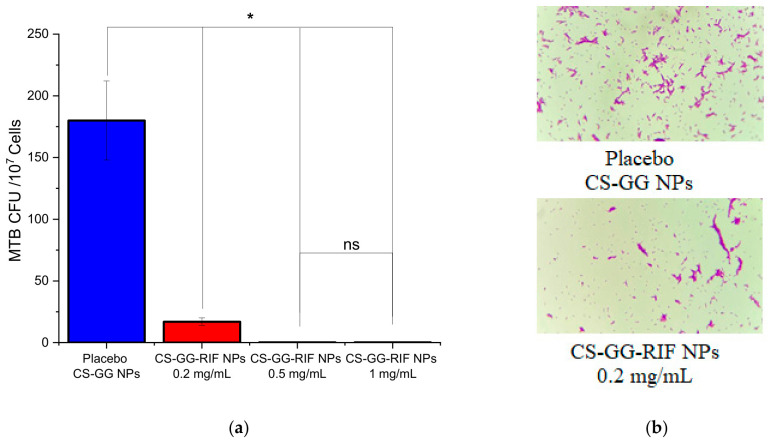
(**a**) Results of antimycobacterial activity test of the nanoparticles on the growth of H37Rv on Löwenstein–Jensen nutrient-dense medium. “*” indicates *p* < 0.05, ns—denotes no significant difference. Data are presented as means ± standard errors of the means (*n* = 3). (**b**) Microphotographs of MTB H37RV growth on the samples, stained using the Ziehl–Neelsen method.

The antituberculosis activity of nanoparticles was tested on H37Rv strains. Mycobacterial growth was studied in Löwenstein–Jensen medium. The antimycobacterial activity of the nanoparticles was investigated at rifampicin concentrations of 0.2 mg/mL, 0.5 mg/mL, and 1 mg/mL. As a control, we studied placebo chitosan–gellan gum nanoparticles without the drug. Samples in which mycobacterial growth was observed were stained using the Ziehl–Neelsen method, and smears were examined under a microscope. The resulting microscopic images were analyzed using the ImageJ software, and the number of colony-forming units (CFUs/10^7^ cells) was calculated. The results are shown in Figure 11.

As shown in the graph, the addition of nanoparticles with rifampicin reduced the growth of mycobacteria compared with the group without the drug (placebo CS-GG NPs). As the concentration of rifampicin increased, the suppression of mycobacterial growth also increased. At concentrations of 0.5 mg/mL and 1 mg/mL, the number of CFUs decreased significantly, confirming the high efficacy of rifampicin in the nanoparticles. Figure 11b shows micrographs of smears stained using the Ziehl–Neelsen method, which also indicated differences in MTB growth. Thus, the minimum inhibitory concentration of rifampicin in CS-GG-RIF nanoparticles for the suppression of *Mycobacterium tuberculosis* growth was determined to be 0.5 mg/mL. It is confirmed that rifampicin loaded in chitosan–gellan gum nanoparticles retained its antituberculosis activity and effectively inhibited the growth of mycobacteria.

## 4. Conclusions

Using the polyelectrolyte complex coacervation method, chitosan–gellan gum-based nanoparticles were produced and effectively loaded with the antituberculosis drug rifampicin. The Taguchi method allowed the synthesis parameters to be optimized and nanoparticles to be produced with specified dispersion characteristics (a minimum size of 153 ± 3 nm, a polydispersity of 0.212 ± 0.021 and a zeta potential of 22.94 ± 1.30 mV), while the chemical structure of the drug remained intact. The polymer matrix ensured the sustained release of rifampicin under in vitro conditions, with the rate of release depending on the pH of the medium. Chitosan and gellan gum nanoparticles demonstrated high mucoadhesive activity towards the lung tissue, confirming their potential as an effective mucoadhesive platform for drug delivery. Rifampicin loaded into the chitosan–gellan gum complex did not lose its ability to inhibit the growth of *Mycobacterium tuberculosis*, particularly with regard to the H37Rv strain. Thus, the chitosan–gellan gum nanoparticles represent a promising platform for creating nanoparticles with adjustable sizes, pronounced mucoadhesive properties, and the possibility of controlled drug release.

## Figures and Tables

**Table 1:** Taguchi L9 experimental scheme for the production of CS-GG-RIF NPs.

Name	Unit	Level 1	Level 2	Level 3
A: GG/CS ratio		1/9	4/6	7/3
B: RIF/polymer ratio		1/3	1/1	3/1
C: temperature	°C	25	40	60
D: volume of ethanol	mL	5	10	15

**Table 2:** Polysaccharide nanoparticles for rifampicin delivery.

Polysaccharide Nanoparticles	Size by DLS Measuring	Encapsulation Efficiency	Method for Producing Polymeric NPs	Release Behavior	Mucoadhesive Properties	Ref.
Alginate–chitosan	Not measured	42–55%	Ionotropic gelation method	At pH 7.4—100% in 8 h At pH 1.2—15–20% in 5 h	Not studied	[43]
Chitosan	10 nm	43%	Ionic gelation method	At pH 4—40% after 24 h At pH 8—70% after 24 h	Not studied	[44]
Chitosan	386 ± 9 nm	20%	Ionotropic gelation method	At pH 1.2—20% after 24 h At pH 7.4 and 6.8—2% after 24 h	At pH 5.5 77% after 4 h At pH 6.8 30% after 4 h	[30]

**Table 3:** Physicochemical characteristics of the produced CS-GG-RIF NPs.

#	GG/CS Ratio	RIF/Polymer Ratio	Temperature (°C)	Vol. of EtOH (mL)	Average Diam. (nm)	Average PDI	Zeta Potential (mV)	EE (%)	NP Yield (%)
1	1/9	3/1	60	15	162 ± 2	0.291 ± 0.022	21.38 ± 0.14	35	19
2	7/3	3/1	40	5	836 ± 19	0.683 ± 0.113	21.42 ± 1.51	73	65
3	7/3	1/1	25	10	167 ± 3	0.291 ± 0.027	17.80 ± 0.72	26	31
4	4/6	3/1	25	10	423 ± 9	0.567 ± 0.039	23.95 ± 1.51	25	25
5	1/9	1/3	25	5	326 ± 1	0.267 ± 0.011	31.65 ± 0.13	44	15
6	7/3	1/3	60	10	194 ± 3	0.221 ± 0.014	24.57 ± 0.72	30	20
7	4/6	1/3	40	15	151 ± 3	0.240 ± 0.004	26.95 ± 1.30	28	40
8	4/6	1/1	60	5	538 ± 10	0.577 ± 0.142	32.48 ± 0.53	20	28
9	1/9	1/1	40	10	237 ± 3	0.392 ± 0.047	28.26 ± 0.36	28	10

Data are presented as means ± standard deviations (*n* = 3).

**Table 4:** ANOVA results for size, PDI and zeta potential.

Source	Sum of Squares	Degree of Freedom	Mean Square	F-Value	*p*-Value
Size					
Model	3.370 × 10^−6^	6	5.616 × 10^−7^	9.94	0.0942
A—GG/CS ratio	3.256 × 10^−7^	2	1.628 × 10^−7^	2.88	0.2576
B—RIF/polymer ratio	5.708 × 10^−7^	2	2.854 × 10^−7^	5.05	0.1652
D—volume of ethanol	2.842 × 10^−6^	2	1.421 × 10^−6^	25.15	0.0382
Residual	1.130 × 10^−7^	2	5.648 × 10^−8^		
Cor total	3.483 × 10^−6^	8			
PDI					
Model	114.50	2	57.25	5.84	0.0391
B—RIF/polymer ratio	114.50	2	57.25	5.84	0.0391
Residual	58.81	6	9.80		
Cor total	173.31	8			
Zeta potential					
Model	25.84	6	4.31	1.07	0.5556
A—GG/CS ratio	17.47	2	8.74	2.18	0.3146
C—temperature	2.77	2	1.38	0.3450	0.7435
D—volume of ethanol	11.31	2	5.65	1.41	0.4149
Residual	8.02	2	4.01		
Cor total	33.86	8			

**Table 5:** ANOVA results for encapsulation efficiency and nanoparticle yield.

Source	Sum of Squares	Degree of Freedom	Mean Square	F-Value	*p*-Value
Encapsulation efficiency					
Model	2.083 × 10^−8^	6	3.472 × 10^−9^	46.14	0.0214
A—GG/CS ratio	9.699 × 10^−9^	2	4.850 × 10^−9^	64.45	0.0153
B—RIF/polymer ratio	8.285 × 10^−9^	2	4.142 × 10^−9^	55.05	0.0178
C—temperature	2.847 × 10^−9^	2	1.423 × 10^−9^	18.92	0.0502
Residual	1.505 × 10^−10^	2	7.525 × 10^−11^		
Cor total	2.098 × 10^−8^	8			
Nanoparticle yield					
Model	0.0208	4	0.0052	9.81	0.0241
A—GG/CS ratio	0.0179	2	0.0090	16.92	0.0112
D—volume of ethanol	0.0066	2	0.0033	6.21	0.0593
Residual	0.0021	4	0.0005		
Cor total	0.0229	8			

**Table 6:** Criteria for the synthesis of CS-GG-RIF NPs.

Name	Goal	Lower Limit	Upper Limit
A: GG/CS ratio	Is in range	1/9	7/3
B: RIF/polymer ratio	Is in range	1/3	3/1
C: temperature	Is equal to 25 °C	25	60
D: volume of ethanol	Is in range	5	15
Size	Minimize	151	836
PDI	Minimize	0.221	0.683
Zeta potential	Maximize	17.80	32.48
Encapsulation efficiency	Maximize	20	73
NPs’ yield	Maximize	10	65

**Table 7:** Predicted and experimental results for CS-GG-RIF NPs.

	Size (nm)	PDI	Zeta Potential (mV)	Encapsulation Efficiency (%)	NPs’ Yield (%)
Predicted	149 ± 2	0.247 ± 0.579	21.55 ± 4.6	25	48
Experimental	153 ± 3	0.212 ± 0.021	22.94 ± 1.30	39	38
Error %	3	14	6	56	21

Data are presented as means ± standard deviations (*n* = 3).

**Table 8:** Coefficients of determination (R^2^) for rifampicin release models from CS-RIF nanoparticles.

pH	Zero-Order	First-Order	Higuchi	Korsmeyer–Peppas
1.2	0.0733	0.7482	0.2519	0.9149
6.8	0.5173	0.9802	0.7867	0.9462
7.4	0.4227	0.9976	0.7073	0.9705

## Data Availability

The original contributions presented in this study are included in this article. Further inquiries can be directed to the corresponding author.

## Abbreviations

The following abbreviations are used in this manuscript:

TB	Tuberculosis
MTB	*Mycobacterium tuberculosis*
CS	Chitosan
GG	Gellan gum
NPs	Nanoparticles
RIF	Rifampicin
PDI	Polydispersity index
EE	Encapsulation efficiency
TGA	Thermogravimetric analysis
DSC	Differential scanning calorimetry
FTIR	Fourier-transform infrared spectroscopy
ANOVA	Analysis of variance
FITC	Fluorescein isothiocyanate
TEM	Transmission electron microscopy
